# Therapies Targeting Epigenetic Alterations in Acute Kidney Injury-to-Chronic Kidney Disease Transition

**DOI:** 10.3390/ph15020123

**Published:** 2022-01-20

**Authors:** Fumiaki Tanemoto, Imari Mimura

**Affiliations:** Division of Nephrology and Endocrinology, The University of Tokyo Graduate School of Medicine; Tokyo 113-0033, Japan; f.tanemoto.18@gmail.com

**Keywords:** AKI-to-CKD, acute kidney injury, chronic kidney disease, histone, hypoxic memory, histone acetylation, histone methylation, DNA methylation, chromatin conformational changes

## Abstract

Acute kidney injury (AKI) was previously thought to be a merely transient event; however, recent epidemiological evidence supports the existence of a causal relationship between AKI episodes and subsequent progression to chronic kidney disease (CKD). Although the pathophysiology of this AKI-to-CKD transition is not fully understood, it is mediated by the interplay among multiple components of the kidney including tubular epithelial cells, endothelial cells, pericytes, inflammatory cells, and myofibroblasts. Epigenetic alterations including histone modification, DNA methylation, non-coding RNAs, and chromatin conformational changes, are also expected to be largely involved in the pathophysiology as a “memory” of the initial injury that can persist and predispose to chronic progression of fibrosis. Each epigenetic modification has a great potential as a therapeutic target of AKI-to-CKD transition; timely and target-specific epigenetic interventions to the various temporal stages of AKI-to-CKD transition will be the key to future therapeutic applications in clinical practice. This review elaborates on the latest knowledge of each mechanism and the currently available therapeutic agents that target epigenetic modification in the context of AKI-to-CKD transition. Further studies will elucidate more detailed mechanisms and novel therapeutic targets of AKI-to-CKD transition.

## 1. Introduction

Chronic kidney disease (CKD) is defined as persistent abnormalities of the kidney structure or renal function [1]. It is a very common condition caused by various kidney diseases with a reported prevalence of more than 10% worldwide [2]. CKD is a significant health problem as an independent risk factor for cardiovascular disease and death [1]. An increasing number of patients have progressed to end-stage renal disease (ESRD) requiring renal replacement therapy. Limited accessibility to treatment is also an issue in many countries of the world [1]. The initial injury may affect various parts of the kidney including the glomerulus, tubules, and interstitium, depending on the underlying cause of CKD. The final pathophysiology of progressive CKD results in tubulointerstitial fibrosis, which is regarded as the final common pathway in the progression to ESRD [3,4]. As with other organs [5], there have been no effective practical therapies for renal fibrosis; therefore, the progression of CKD is still characterized by irreversible pathological processes [6].

In contrast to CKD, acute kidney injury (AKI) is defined as a sudden loss of renal function, which is also a very common condition with high morbidity and mortality during the acute phase [7,8]. Decreased renal function is often associated with a lack of renal blood flow and local or systemic inflammation [9]. The most common causes of AKI include ischemia/reperfusion (I/R) injury, sepsis, and exposure to nephrotoxic agents, such as cisplatin [10,11].

AKI was previously recognized as a transient event, which resolved spontaneously, resulting in no consequences [12]. However, emerging evidence from epidemiological studies and meta-analyses suggests the existence of a causal relationship between AKI episodes and subsequent progression to CKD [12,13,14,15]. Moreover, animal studies indicate that maladaptive repair following recovery from AKI results in decreased kidney function and the development of renal fibrosis, which supports the existence of AKI-to-CKD transition [12,16]. Today, the mechanism that drives the transition from AKI to CKD is considered to be mediated by an interplay among multiple factors including tubular epithelial injury, endothelial dysfunction, interstitial inflammation, and fibrosis; however, it is still being debated [16,17]. Recently, evidence suggests that epigenetic mechanisms, regulating gene expression and the downstream cellular response, are largely involved in AKI-to-CKD transition [18]. Because of its reversibility, epigenetic modifications have the potential to be a source of novel therapeutic targets for AKI-to-CKD transition [18] and have attracted many researchers to this rapidly developing field.

In this review, we will first summarize the current concepts and pathophysiology of AKI-to CKD transition. Then, the latest evidence for epigenetic modifications contributing to the pathogenesis of AKI-to-CKD transition and their potential role as novel therapeutic targets are discussed in detail.

## 2. Current Overview of the Mechanisms Involved in AKI-to-CKD Transition

### 2.1. The Pathophysiology of AKI-to-CKD Transition

Recent epidemiological studies and meta-analyses strongly support the existence of a causal relationship between AKI and the subsequent transition to CKD; however, the pathophysiological mechanism has not been fully revealed [16,17]. Evidence gathered thus far derives from animal studies of kidney injury [9].

**Figure 1:**
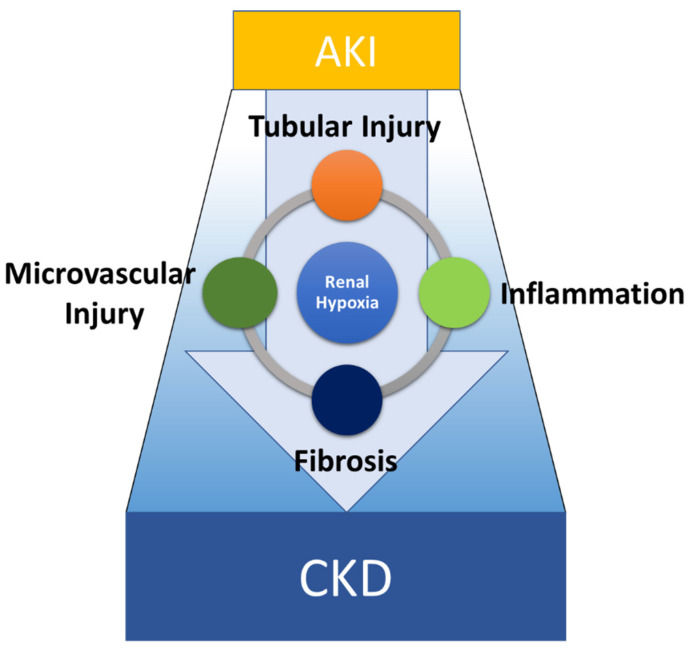
Conceptual diagram of the pathogenesis of AKI-to-CKD transition. AKI-to-CKD transition is mediated by the interplay among multiple components of the kidney including tubular epithelial cells, endothelial cells, pericytes, inflammatory cells, and myofibroblasts. Four main pathologies including tubular injury, microvascular injury, inflammation, and fibrosis, commonly associated with renal hypoxia, contribute to disease progression.

The kidneys consist of various types of differentiated cells with specific functions that contribute to the maintenance of internal homeostasis. Various alterations in kidney structure following AKI, including nephron loss, incomplete tubular repair, vascular rarefaction, interstitial inflammation, and shifts in interstitial cellular composition, interact with one another to contribute to the progression of CKD [9] (Figure 1).

Tubular epithelial cells (TECs) are the most abundant cell type in the kidney. They are considered to be the primary target of AKI because of their susceptibility to various types of stress, such as ischemia-induced hypoxia and nephrotoxic substances [19,20]. Hypoxia is an important factor in the pathogenesis of various kidney diseases, because the kidney is physiologically in a hypoxic state that is maintained by its diffusional oxygen shunt between the arterial and venous vessels [6,12]. This enables the kidney to extract no more than 10% of available oxygen and thereby render it intrinsically susceptible to further hypoxic stress [6,12]. Consequently, injured TECs change their phenotype to produce various bioactive substances that drive interstitial inflammation and fibrosis, which contributes to the progression of CKD [21]. Endothelial cells are also important players in the response to AKI. Rarefaction of the peritubular capillaries occurs following AKI insult and results in renal hypoxia, which and has an important role in the subsequent progression to CKD [12]. Although the molecular mechanism responsible for capillary rarefaction has not been elucidated [21], decreased expression of vascular endothelial growth factor (VEGF), an endogenic angiogenic factor produced by TECs, and detachment of pericytes, which are located close to endothelial cells and play a role in maintaining vascular stability, occur after AKI and may contribute to this mechanism [13,17]. Subsequent tissue hypoxia induces sterile inflammation and fibrosis. Tubulointerstitial fibrosis aggravates hypoxia because of the further loss of capillaries and increased physical distance between resident TECs and capillaries, which is a vicious cycle that results in the further progression of CKD [12,13]. Inflammatory cells, including resident and infiltrating immune cells, such as macrophages and neutrophils, contribute to disease progression through cross-talk with other cell types [17,22]. Myofibroblasts are activated forms of fibroblasts and the principal cell type that produces extracellular matrix (ECM) to form a fibrotic lesion [5,23]. They are predominantly derived from resident fibroblasts and pericytes [2]. Although pericyte detachment and capillary rarefaction may be caused by AKI, pericyte loss can also trigger endothelial damage and capillary rarefaction, resulting in TEC injury and fibrosis [17,24].

The features described above represent only a portion of the emerging evidence that demonstrate a contribution of various factors in AKI-to-CKD transition. Further details of the underlying mechanisms will provide prospective targets for therapeutic intervention.

### 2.2. TECs Are a Primary Target of AKI and a Driver of Inflammation and Fibrosis

As mentioned above, TECs, which occupy most of the renal cortex, have a large influence on the pathophysiology of AKI-to-CKD transition. Here, we focus on their physiological response to ischemic AKI insult.

When TECs are exposed to mild ischemia of reversible hypoperfusion, the subsequent reduction in renal function is not accompanied by parenchymal injury [7]. However, once the ischemic time exceeds a certain threshold, ischemic tubules undergo acute tubular necrosis (ATN) [25]. In the acute phase, necrotic tubular cells release signals that activate pattern recognition receptors on resident immune cells in the interstitium. This results in a local inflammatory response that aggravates tubular necrosis through an influx of neutrophils and macrophages. An auto-amplification loop forms known as necroinflammation, which is resolved by various counter-regulators [7,26,27].

Once TECs become necrosis, they are irreversibly lost during acute necroinflammation. Currently, there are two proposed physiological responses to injured tubules: limited regeneration by renal progenitors and polyploidization of remnant TECs [28]. Renal progenitors are undifferentiated cells that are scattered among the differentiated TECs, primarily in the S3 segment (the most vulnerable to ischemic insult) during a healthy state. They are relatively resistant to death and can regenerate fully differentiated TECs following necrotic injury [29,30]. These renal progenitors, rather than the majority of remnant TECs, are considered a source of TEC regeneration following AKI. This is supported by the result of lineage tracing studies [31]. On the other hand, the majority of remnant tubular cells are thought to enter the cell cycle and undergo endoreplication-mediated hypertrophy, known as polyploidization, which is observed in other tissues and organs [17]. This increase in the gene copy number without cell division quickly meets the increased functional demand for a large metabolic output [17,32]. The complete loss of renal progenitors by severe injury results in irreversible nephron loss and the total number is a key determinant of the long-term prognosis following AKI [31]. Although polyploidization of TECs can augment the functional capacity to compensate for nephron loss to some extent, it cannot regenerate parenchymal loss. Therefore, undergoing polyploidization results in a predisposition to subsequent CKD [17].

Although this dynamic physiological repair mechanism following tubular injury explains the background pathology of AKI-to-CKD transition in part particularly for moderate to severe AKI, this mechanism does not encompass all of the factors associated with TECs contributing to pathogenesis. Phenotypic changes of injured TECs are also considered an important factor that contributes to AKI-to-CKD progression. Injured TECs have been shown to exhibit an inflammatory phenotype that mediates the immune response directly by producing inflammatory cytokines or indirectly through infiltrating leukocytes [33]. Moreover, after severe or recurrent injury, TECs undergo phenotypic changes that promote gene expression and the production of profibrotic factors [33]. Cell cycle arrest of TECs, which may be related to maladaptive repair, may be related to the inflammatory phenotype, ECM deposition, and subsequent TECs acquisition of a pro-fibrotic and senescent phenotype [17,34].

### 2.3. Molecular Mechanisms of AKI-to-CKD Transition: Insights from Omics Approaches

Recent studies examined the time-course of comprehensive gene expression associated with AKI-to-CKD transition, including single-cell analyses [35,36,37,38,39]. Liu et al. conducted a comprehensive gene expression analysis at multiple time points by bulk RNA-sequencing (RNA-seq) of whole kidney using a murine bilateral ischemia/reperfusion injury (IRI) model. This is a widely used experimental animal model in which renal arteries are clamped for a certain period of time to mimic ischemic AKI [35]. They showed that there were several temporal-specific patterns of gene expression related to function including tubular injury and repair, fibrosis, and the inflammatory response [35]. Understanding the mechanisms underlying the regulation of gene expression including epigenetic aspects may lead to the development of target-specific novel therapies with timely interventions. Cippa et al. performed time-course transcriptional profiling of human kidney injury progression using biopsy samples of kidney allografts at multiple timepoints, in which ischemia/reperfusion injury occurred during kidney transplantation contributed to chronic allograft dysfunction [37]. The results showed a strong concordance with previous mouse IRI results, suggesting conservation of most injury-induced gene regulatory responses across species and the validity of using mouse models to further understand human disease [37]. There have also been joint profiling studies of chromatin accessibility and gene expression of healthy kidneys in mice [40] and humans (specimens after mass nephrectomy) [41]. These combined the results of single-nucleus RNA-seq and single-nucleus ATAC-seq (assay for transposase-accessible chromatin with high-throughput sequencing), a technique that captures open chromatin sites [40,41]. With the recent advances in sequence technology, the details of the epigenetic regulatory mechanism of gene expression associated with AKI-to-CKD transition will be further elucidated.

Recent advances in other omics technologies, such as mass spectrometry-based proteomics and metabolomics, has contributed to an understanding of AKI-to-CKD transition. These include detecting novel biomarkers that detect or predict the prognosis of CKD and pathways that contribute to CKD, which could be novel therapeutic targets for intervention [42]. Recently, mass spectrometry-based proteomics was performed in mouse IRI models, which detected the downregulation of phosphatase and tensin homolog on chromosome ten (PTEN) in mice with maladaptive repair. This protein may represent a novel therapeutic target [43]. Also, liquid chromatography–mass spectrometry (LC–MS)-based metabolomics revealed endogenous renal metabolism in mice, in which the mitochondrial biogenesis regulator, PGC1α (peroxisome proliferator-activated receptor γ coactivator 1-α), was a key determinant of kidney recovery from AKI through the regulation of nicotinamide adenine dinucleotide (NAD) biosynthesis [44,45]. The sirtuins (SIRT) are a family of well-conserved NAD^+^-dependent histone deacetylases [46]. SIRT3, which is highly expressed in renal proximal TECs and widely distributed in mitochondria, plays an important role in regulating mitochondrial dynamics and metabolism [47]. SIRT3 maintains mitochondrial homeostasis against IRI stimulation, suggesting that it may be a suitable target for therapeutic intervention of kidney injury [46,48].

Recent advances in collective analyses of cells, tissues, and organs will enable us to reveal further details of the molecular mechanisms involved in AKI-to-CKD pathology from a new perspective and in an unbiased manner [42,49]. The effective use of omics technology is expected to provide a better understanding of the details of epigenetic alteration.

## 3. Epigenetics of AKI-to-CKD Transition

### 3.1. Epigenetics Overview

Epigenetics refers to the mechanism of reversible and heritable changes in gene expression that are not caused by alterations in the nuclear DNA sequence itself [50].

**Figure 2:**
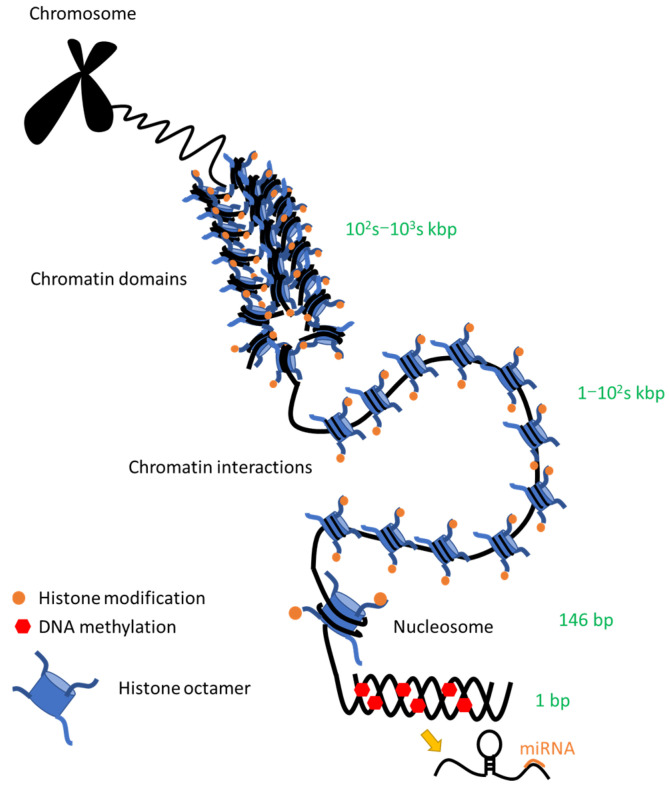
Chromatin structure in the nucleus. DNA strands are packaged into higher order structures called chromatin in the nucleus. A nucleosome is the structural unit of chromatin, which consists of 146 base pairs of negatively charged DNA wound around a positively charged histone octamer containing two each of four core histones: H2A, H2B, H3 and H4 [57]. The chromatin condition is fundamental to the control of gene expression [58]. Highly compacted heterochromatin effectively shuts off all gene expression, whereas loosely configured euchromatin provides accessibility to transcription factors and RNA polymerases and promotes gene transcription [57]. Even in the latter state, the degree of relaxation is not uniform, and genes that are highly actively transcribed are in a more relaxed region. The chromatin structure and epigenetic alterations discussed in this review are shown here with their scales [59]. Chromatin interactions include long-range interactions between regulatory elements, such as enhancers and their target genes. Chromatin domains include topologically associating domains (TADs), which are reviewed later.

Epigenetic features include modifications of DNA or histone proteins, or RNA interference mediated by non-coding RNAs [51] that regulate gene expression by changing the chromatin structure or accessibility of genetic loci by transcriptional machinery [52] so that seemingly identical genes exhibit different phenotypes in a temporally and spatially regulated manner [53] (Figure 2). These modifications are stable during cell division and may be influenced by environmental factors, aging, or disease [51], and are then stored as an epigenetic memory [54]. For example, altered epigenetic markers at an earlier age not only trigger acute adaptive responses but can also predispose an organism to late-onset diseases following secondary triggers [55]. Epigenetic alterations are also potentially reversible and the reversal of these alterations represents promising targets for the prevention and treatment of diseases promoted by epigenetic mechanisms [56].

In the process of transcriptional regulation by chromatin structural changes, chemical modifications of DNA (DNA methylation) or histone proteins function as marks for distinguishing areas that should or should not be transcribed [57]. Specific enzymes called “epigenetic writers” catalyze additional reactions on these epigenetic marks, which may be removed by enzymes called “epigenetic erasers” [51]. These marks are recognized by functional proteins called “epigenetic readers”, and their consequent biological pathways modulate chromatin accessibility and regulate gene expression.

### 3.2. Involvement of Epigenetics in the Pathogenesis of AKI-to-CKD Transition

As reviewed in the previous chapter, hypoxia is a central player that contributes to the pathophysiology of AKI-to-CKD transition (Figure 1). During the course of AKI-to-CKD transition, hypoxia-induced epigenetic changes are recorded in the cell that have a long-term effect, known as “hypoxic memory” [60]. These refer to epigenetic transcriptional regulations that induce the progression to CKD in the long term following complete recovery from an initial AKI episode [12,61].

Our group previously performed several studies that showed hypoxia-induced epigenetic regulations contribute to the pathogenesis of AKI-to-CKD transition. Using a 3C assay, we found that hypoxia-inducible factor (HIF)-1, a master transcription factor under hypoxia, and lysine-specific demethylase 3A (KDM3A) conjugately regulated the expression of a down-stream target gene, solute carrier family 2A3 (SLC2A3), through a chromosome conformational change [53,62]. Under normoxia, a 35 kb upstream region of the SLC2A3 promoter formed a loop via HIF1, which disappeared when HIF1 was knocked down using siRNA. Under hypoxia, in addition to recruitment of KDM3A to the SLC2A3 loci, chromosome conformational changes upregulated SLC2A3 expression [53,63]. We also exposed cultured tubular cells to hypoxia and identified HIF-1 downstream targets in a genome-wide analysis of HIF-1 binding sites [64]. Combining the results of RNA-seq and chromatin immunoprecipitation (ChIP)-seq [65], 44 long non-coding RNA (lncRNA) were identified that were shared by multiple tubular cell lines. The expression of novel lncRNA, DARS-AS1 (aspartyl-tRNA synthetase anti-sense 1), was upregulated only under hypoxia and HIF-1 was bound to its promoter region, which included two hypoxia-responsive elements [60,64]. Functionally, DARS-AS1 plays an important role in inhibiting apoptosis in renal tubular cells [64].

These two epigenetic regulatory events are located downstream of HIF-1. The pathogenesis of AKI-to-CKD transition includes various phenotypic alterations in a variety of cell types. Regardless of whether it goes through hypoxia or not, epigenetic regulation of gene expression is considered an important process in the pathogenesis of AKI-to-CKD.

### 3.3. Histone Modification

#### 3.3.1. Histone Acetylation

1. Basic Mechanisms of Histone Acetylation

**Figure 3:**
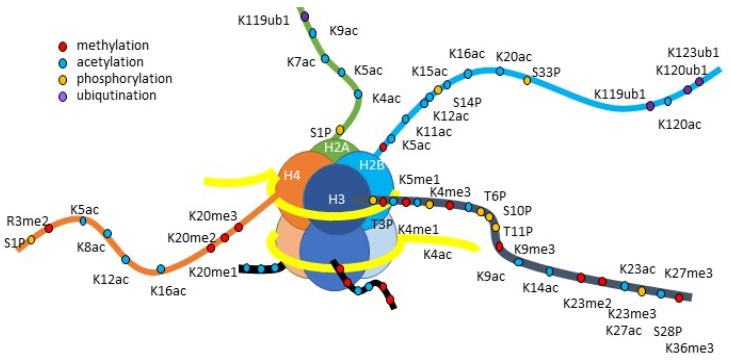
Histone structure and possible sites to be modified. Histones are a main component of chromatin as well as DNA. They are comprised of a globular domain and a flexible and positively charged N-terminal called a “histone tail” that protrudes from the nucleosomes [73]. The amino acid residues located in the histone tail are subject to post-translational modifications [57]. These modifications either positively or negatively regulate transcription by modulating the chromatin structure or providing binding sites for the transcriptional machinery [51].

Histone acetylation regulates gene expression in multiple ways. The addition of an acetyl group neutralizes the positive charge of lysine, which relaxes the chromatin structure to form an open chromatin configuration and allows transcription factors more access to their target genes [66]. Acetylated lysine residues also function as docking sites for many transcriptional activators, such as bromodomain proteins [67] (Figure 3).

Histone acetylation is reversible and fairly dynamic. It is catalyzed by histone acetyltransferases (HATs) and deacetylation is catalyzed by histone deacetylases (HDACs) [51]. The balanced actions of HATs and HDACs are key regulatory mechanisms for gene expression involving various developmental processes and diseases [67]. HATs include some groups classified by amino acid sequences and conformational homology, such as GNAT, MYST, and the CBP/p300 subfamilies [68]. In mammals, 18 HDAC proteins have been identified, which are divided into the following four groups: class I includes HDACs 1, 2, 3, and 8; class II includes HDACs 4, 5, 6, 7, 9, and 10; class III includes silencing proteins, sirtuins (SIRT1-7); and class IV includes HDAC11 [51]. They depend on zinc for their catalytic activity, except for the class III HDACs, which require NAD^+^ for catalytic activity [67].

Readers of epigenetic modifications commonly include one or more than one effector domains that recognize target modifications of histones and DNA [69]. The bromodomain is an evolutionarily conserved motif of 110 amino acids contained in proteins interacting with chromatin, including transcription factors, HATs, and nucleosome remodeling complexes [70]. Bromodomain (BRD) proteins function as readers of histone acetylation by facilitating acetylation-dependent recruitment of transcriptional regulator complexes [71]. BRD proteins containing two N-terminal bromodomains and an extra-C terminal domain are classified as BET (bromodomain and extra-terminal domain) family members, which consists of four mammalian members: BRD2, BRD3, BRD4, and BRDT [72].

2. Histone Acetylation in AKI-to-CKD Transition

The level of histone acetylation was shown to change globally during the course of ischemic AKI. In murine IRI, H3 acetylation was transiently decreased in proximal tubular cells, probably resulting from reduced HAT activity during ischemia [74]. Decreased H3 acetylation was restored after 24 h of reperfusion and accompanied by the induction of a key regulator of renal repair, bone morphogenetic protein 7 (BMP7), which is associated with the selective downregulation of HDAC5 during the recovery phase [74]. However, combined with the results of other similar studies with varying degrees and duration of changes in histone acetylation, these depend on factors such as the intensity of the injury, mouse lineage, and analytical methods [51]. Other nephroprotective genes, such as Klotho and PGC-1a, were also shown to be downregulated by inflammatory cytokine-mediated deacetylation in a folic acid-induced AKI model. Downregulation was prevented by administration of HDAC inhibitors in cultured tubular cells exposed to inflammatory cytokines [75,76].

The degree of H3 histone acetylation was progressively increased over the baseline, together with the induction of inflammatory and pro-fibrotic genes, which is consistent with AKI-to-CKD progression [77]. Moreover, at ten days after unilateral ureteral obstruction (UUO) as a fibrotic kidney model, the level of H3K9 acetylation (H3K9Ac) was globally increased [78]. In a murine model of lipopolysaccharide (LPS)-induced septic AKI, the induction of a HAT, p300/CBP-associated factor (PCAF), was associated with increased acetylation of H3K18 and upregulated inflammatory genes [79]. Conversely, silencing of PCAF resulted in a significant decrease in H3K18Ac and inflammatory factors including VCAM1, ICAM1, and MCP1 in cultured tubular cells [51].

Based on these findings, histone acetylation may be involved in the repression of renoprotective genes in the acute phase and upregulation of inflammatory and profibrotic genes in the chronic phase of AKI-to-CKD transition. In fact, the level of histone acetylation over the course of AKI is heterogeneous and considered to be injury-, time-, and gene-specific [71,80]. With respect to the treatment of AKI and its repair, HDAC inhibitors, HDAC activators, and HAT inhibitors may exert therapeutic effects [51].

- HDAC Inhibitors in AKI-to-CKD Transition

There is evidence to indicate that HDACs have an important role in the pathophysiology of AKI through transcriptional regulation of various genes involved in apoptosis, inflammation, oxidative stress, ATP production, and fibrosis [81]. They regulate pro-inflammatory cytokine release, EGFR (epidermal growth factor receptor), TGF/Smad, NF-κB signaling, and cell cycle arrest [81]. Class I and II HDAC inhibitors are generally protective during experimental renal injury [71]. However, class I HDAC inhibitors have exhibited mixed results in different AKI models, which may result from differences in the time course of tubular repair among AKI models, the structures of administered HDAC inhibitors, and the dose and timing of administration [81]. Class III HDACs, sirtuins, are expressed in different subcellular compartments and play different roles in cellular homeostasis. Many studies have shown that sirtuins are involved in the pathogenesis of AKI through regulation of oxidative stress, apoptosis, inflammation, autophagy, and mitochondrial biogenesis. Overexpression of SIRT1, SIRT3, and SIRT6, and the suppression of SIRT7 and SIRT2 are renoprotective [81]. For class IV, HDAC11 gene silencing increased PAI-1 expression, which can induce inflammation and functional impairment following ischemic insult [82]. HDAC11 may represent a new therapeutic target for AKI [81].

Although a variety of HDAC inhibitors have been shown to exhibit anti-fibrotic and anti-inflammatory activity and effectiveness on various renal diseases in animal models [83], their application to human clinical trials has been limited because of their adverse effects, which include hyponatremia, hypokalemia, edema, and blood pressure changes [81,84]. To reduce toxicity and improve clinical efficacy, more specific HDAC inhibitors, rather than pan-HDAC inhibitors or class-specific HDAC inhibitors, need to be developed [81]. Moreover, the precise dosing for the desired effect, the proper timing of therapeutic intervention, and effective drug delivery should all be taken into account for clinical application.

- HDAC Activators in AKI-to-CKD Transition

Resveratrol, SRT-2183, and SRT-1720 are SIRT1 activators, which exert effects on AKI and subsequent repair [51]. Resveratrol exhibits renoprotective effects in cisplatin-induced and septic AKI models [85,86]. Moreover, the administration of SRT-1720 in a murine IRI model induced the proliferation of renal tubular cells and attenuated renal injury [87].

- HAT Inhibitors in AKI-to-CKD Transition

Curcumin, a HAT inhibitor that inhibits p300/CBP, was shown to have protective effects in various AKI models, including cisplatin-induced, LPS-induced AKI, and IRI [88]. However, curcumin has other targets including HDACs, so whether a beneficial effect is due to HAT inhibition is unclear [89].

#### 3.3.2. Histone Methylation

1. Basic Mechanisms of Histone Methylation

Unlike acetylation, histone methylation does not change the electrical charge of the histone proteins. Instead, it provides sites for the binding of transcription regulators associated with either activating or repressing target gene expression, depending on methylation state and position [51,90]. Histone methylation on H3K4, H3K36, and H3K79 is associated with activating gene expression, whereas methylation on H3K9, H3K27, and H4K20 is linked to the repression of transcription [90].

**Figure 4:**
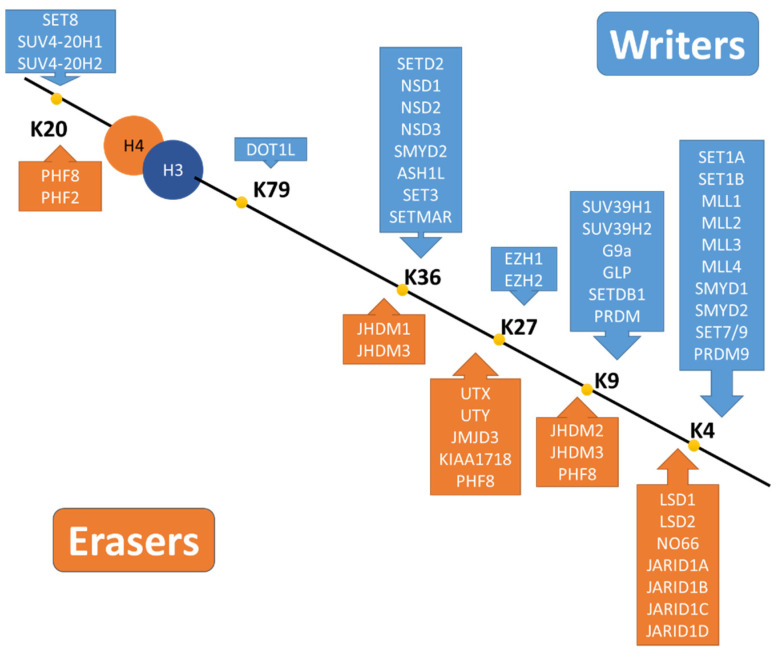
Writers and erasers of histone methylation. Major methylation sites are located in the histone tails of H3 and H4 (H3K4 (histone 3 lysine 4), H3K9, H3K27, H3K36, and H4K20) and also H3K79 in the nucleosome core region may be methylated [93]. Writers and erasers of histone methylation reported in human are listed with their sites of action in this figure [91].

There are many histone methyltransferases (HMTs) that function as “writers” and demethylases as “erasers” for each site of methylation and their actions are precisely balanced as shown in Figure 4 [91]. Since SUV39H1, which contains a catalytic SET domain, was reported as the first identified HMT in 2000 [92], many HMTs have been identified through SET-domain homology searching [91]. Although histone methylation was once thought to be a low plasticity modification, the discovery of novel histone demethylases, including lysine-specific demethylases (LSD1 and LSD2) and Jumonji (JmjC)-domain containing histone demethylases, has changed this view and broadened our repertoire of histone demethylases [51,91]. There are distinct effector proteins that recognize specific methylated lysine residues corresponding to the neighboring amino-acid sequence and methylation state, and these “readers” are reviewed in detail elsewhere [91].

2. Histone Methylation in AKI-to-CKD Transition

To date, the overall pattern of histone methylation during kidney injury has not been analyzed; however, there is information on specific markers [71]. In a fibrotic kidney model, 10 days after UUO, there was an increase in the global kidney H3K9 trimethylation (H3K9me3) [78]. Moreover, both H3K27me3 and H3K4me3 were significantly upregulated in UUO fibrotic kidney models and human CKD kidneys, which were intimately associated with the fibrotic process [78,94]. Furthermore, there are some reports on altered histone methylation for specific genes. Increased H3K4me3 methylation following IRI was closely associated with the upregulation of inflammatory genes (TNF-α, CCL2), pro-fibrotic genes (TGF-β1, type III collagen), and cholesterol regulatory genes (HMGRC), which ultimately leads to a gradual progression to CKD [95,96,97,98,99].

The methylation processes mentioned above are catalyzed by their specific HMTs, EZH2 (for H3K9me and H3K27me), SET7/9 (for H3K4me), and G9a (for H3K9me) [71,95]. Following the occurrence of AKI, they induce their specific histone methylation, which promotes subsequent renal fibrosis [95].

Kidney inflammation and fibrosis were significantly improved after the blocking of these HMTs. The EZH2 inhibitor, 3-deazaneplanocin A (DZNeP), decreased fibrosis in the UUO model, decreasing signaling from several receptors including TGF-β1, EGFR, and platelet-derived growth factor b receptor (PDGFbR), and PTEN, which may exert a therapeutic effect [94]. Our group performed a genome-wide analysis of TECs using RNA-seq in vivo and in vitro and discovered that DZNeP decreased the expression of pro-fibrotic genes and inhibited tubulointerstitial fibrosis in a murine IRI model of AKI-to-CKD progression [100]. The SET7/9 inhibitor, sinefungin, and the G9a inhibitor, BIX01294I, also decreased fibrosis and reduced the levels of H3K4me1 or H3K9me1, respectively, in kidneys from UUO mice [101,102]. Histone methylation and demethylation inhibitors are in clinical trial for some malignant hematologic diseases. However, there is no information on these drugs being examined in the clinic or their effects on clinical kidney injury [71].

#### 3.3.3. Other Histone Modifications

The recent application of mass spectrometry-based proteomics identified novel histone lysine acylation including propionylation, butyrylation, 2-hydroxyisobutyrylation, β-hydroxybutyrylation, malonylation, succinylation, crotonylation, glutarylation, and lactylation [103]. Of these, lysine crotonylation is a recently described posttranslational modification [104], which adds a crotonyl group (CH_3_CH=CHCO_2_) from crotonyl-CoA to lysine residues catalyzed by histone crotonylases [71]. In addition to gene transcription, histone crotonylation has been suggested to have a potential role in spermatogenesis and AKI [71,105]. The detailed mechanisms of this modification still need to be clarified [106].

### 3.4. DNA Methylation

#### 3.4.1. Basic Mechanisms of DNA Methylation

Generally, methylation of the promoter region is associated with repressed gene expression, which contributes to physiological silencing to prevent chromosomal instability caused by the transcription of repetitive sequences [57], whereas methylation of a gene body tends to be associated with active transcription [107]. CpG promoter methylation may suppress gene expression by the following two mechanisms: direct inhibition of the recruitment of transcription factors to the promoter, or indirectly through the function of recruited DNA methylation readers to form a repressor complex [51]. The susceptibility of the methylation of the promoter region depends on whether the region contains CpG islands. They are rarely methylated, whereas CpG sites present in promoter regions other than CpG islands are highly methylated and gene expression is repressed. Over two thirds of mammalian promoters have CpG islands and almost all of housekeeping genes contain CpG islands [108].

- Writers and Erasers of DNA Methylation

DNA methylation is catalyzed by DNA methyltransferases (DNMTs). The DNMT family consists of DNMT1, DNMT2, DNMT3A, DNMT3B, and DNMT3-like (DNMT3L) [109]. Among these, DNMT1, DNMT3A, and DNMT3B are major DNMTs essential for animal development [51]. DNA methylation includes the establishment (de novo DNA methylation) catalyzed by DNMT3A and DNMT3B and maintenance during DNA duplication, which is catalyzed by DNMT1 [108].

**Figure 5:**
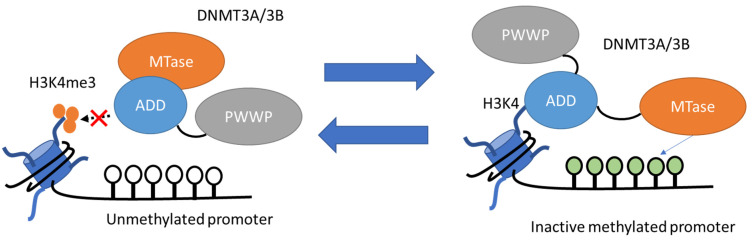
The mechanism of de novo DNA methylation. (**Left**): In the presence of H3K4me3, the ADD domain of DNMT3A/3B cannot bind to H3K4 and instead binds to the MTase domain, which leads to auto-inhibition of enzymatic activity. (**Right**): In the absence of H3K4me3, the ADD domain binds to unmethylated H3K4 and releases the MTase domain with catalytic activity, which leads to methylation of the target promoter region.

The mechanism of de novo DNA methylation is shown in Figure 5 [108]. DNMT3A and DNMT3B contain a highly conserved DNMT domain known as the MTase domain, and two chromatin reading domains known as ATRX-DNMT3-DNMT3L (ADD) and PWWP [108]. During the process of de novo methylation, the ADD domain binds to unmethylated H3K4 and subsequently releases the MTase domain, which catalyzes methylation of the target promoter region [110]. On the other hand, in CpG islands, promoters of actively transcribed genes typically contain rich H3K4me3 marks [111]. The ADD domain cannot bind to H3K4 and instead binds to the MTase domain, which results in auto-inhibition of the enzyme activity [108].

During the process of maintaining DNA methylation, DNMT1 in combination with UHRF1, a member of the ubiquitin-like with PHD and ring finger domain-containing (UHRF) protein family [51], recognizes hemimethylated CpG dinucleotides at replication forks and methylates the daughter DNA strand [112]. Thus, the absence or inhibition of DNMT1 or UHRF1 results in passive DNA demethylation.

DNA methylation was once considered a stable modification down to daughter cells [51]. However, it has become clear that there is a mechanism of active DNA demethylation through which mammalian methylated DNA can still be reversed to an unmodified state [113]. Proteins of the ten-eleven translocation (TET) family, including TET1, TET2, and TET3, can mediate the consecutive oxidation of 5mC to 5-hydroxymethylcytosine (5hmC), 5-formylcytosine (5fC), and 5-carboxylcytosine (5caC) [113]. All of the oxidized forms are not recognized by a maintenance mechanism of DNA methylation mentioned above resulting in DNA demethylation during replication. As for 5fC and 5caC, demethylation can also be mediated through base removal by thymine DNA glycosylase (TDG) followed by base excision repair (BER) [108].

#### 3.4.2. DNA Methylation in AKI-to-CKD Transition

In mouse IRI kidneys, the global level of 5hmC was decreased, whereas that of 5mC was unchanged. This was accompanied by downregulation of TET1 and TET2, but not of TET3 [114]. This decrease in 5hmC enrichment was observed in the promoter regions of the pro-inflammatory genes, Cxcl10 and Ifngr2, which were associated with their increased expression [114]. The transient decrease in genome-wide and CpG methylation continued for up to 7 days and was associated with the downregulation of gene expression after IRI for some methylated genes. This suggests that this promoter methylation contributes to the persistent alteration of gene expression [115]. Moreover, hypermethylation of renoprotective genes, such as Klotho, erythropoietin, and Ras GTPase activating-like protein 1 (RASAL1), are involved in the progression of CKD after AKI, which may promote fibrosis [95,116,117,118].

DNMTS inhibitors including 5-azacytidine (5-aza) and 5-aza-2-deoxycytidine (decitabine), and hydralazine, which exhibit demethylating activity probably by increasing TET3 expression, prevent AKI-to-CKD progression by reducing methylation of the downregulated genes mentioned above to restore their expression [95,116,119]. RASAL1, a renoprotective gene that inhibits fibroblast activity, is downregulated by promoter methylation and causes the activation of fibroblasts, an increase in TGF-β and type I collagen secretion, and subsequent progression of interstitial fibrosis [95]. In RASAL1-deleted mice, demethylation treatment effectively restored RASAL1 expression and successfully delayed AKI-to-CKD transition [120]. RASAL1 methylation was not observed during mild and reversible injury, and the level of its methylation was positively correlated with renal fibrosis after severe and irreversible injury [95]. Low-dose hydralazine induces TET3 expression, which promotes demethylation of the RASAL1 promoter and results in the inhibition of renal fibrosis after ischemic injury [119]. Hydralazine is an antihypertensive agent that has been used since the 1950s and is now mostly used for resistant hypertension and hypertension during pregnancy [121]. With the advantage of its relatively benign adverse effects and established safety profile, it is now being investigated for its potential role in epigenetic regulation as a potential therapeutic agent to prevent CKD progression [122].

The effects of individual epigenetic drugs on the pathology of AKI-to-CKD transition reviewed above are summarized in Table 1 [51,71,95].

### 3.5. Non-Coding RNAs

#### 3.5.1. Basic Mechanisms of Non-Coding RNAs

Non-coding RNAs, mainly microRNAs and long non-coding RNAs (lncRNAs), play a role as epigenetic regulators [142]. Non-coding RNAs may be divided into small non-coding RNAs (<200 nucleotides) and long non-coding RNAs (>200 nucleotides), both of which regulate gene expression through their respective mechanisms [54].

Small non-coding RNAs may be divided into three classes: microRNAs, small interfering RNAs (siRNAs), and piwi-interacting RNAs (piRNAs) [54]. MicroRNAs, the most intensively studied RNAs, contain 21 to 25 nucleotides and function as post-transcriptional inhibitory regulators of gene expression [51]. MicroRNAs bind to the 3′ untranslated region (UTR) of their target gene mRNA and exert inhibitory effects by either inducing mRNA degradation or, more commonly, inhibiting the translation of mRNA into protein [51,143]. The siRNAs are involved in heterochromatin formation and chromosome condensation through the RNA-induced transcriptional silencing complex and piRNAs contribute to posttranscriptional silencing of transposons [54,144,145].

Long non-coding RNAs (lncRNAs) can regulate gene expression positively or negatively at both transcriptional and post-transcriptional levels [51,146]. They participate in various physiological processes including cell proliferation, cell cycle progression, differentiation, apoptosis, and inflammation [95]. During transcriptional regulation, lncRNAs function by the following mechanisms: recruiting transcriptional regulators, acting as decoy factors by binding transcription factors or other proteins away from DNA, guiding chromatin-modifying enzymes to their target gene, or serving as a scaffold to recruit multiple factors to form transcription regulatory complexes [51,147].

#### 3.5.2. Non-Coding RNAs in AKI-to-CKD Transition

Both microRNAs and lncRNAs are important epigenetic regulators during the course of AKI and the subsequent repair process [51].

MicroRNAs play an important role in regulating various cellular and physiological processes including cell proliferation, differentiation, organ development, and cell death, and involve the pathogenesis of a variety of human diseases [51]. By binding to target genes, they regulate the inflammatory response, cell cycle, and apoptosis during the injury and repair phases of AKI, thereby affecting the subsequent transition to CKD protectively or pathogenically [95]. Protective microRNAs that improve kidney function by reducing the inflammatory response and fibrosis include miR-17-5p, miR-27a-3p, miR-126, miR-205, and miR-688 [148,149,150,151,152]. On the other hand, pathogenic microRNAs that promote CKD progression include miR-24, miR-150, miR-181a, miR-494 and miR-687 [153,154,155,156,157]. MiR-21 is generally protective but exhibits a bilateral character in that mild upregulation inhibits fibrosis and the inflammatory response, whereas sustained upregulation promotes tubulointerstitial fibrosis following AKI [158].

LncRNAs also play various roles in regulating different physiological processes, such as cell cycle, cell proliferation, apoptosis, differentiation, and inflammation. They participate in AKI-to-CKD transition [95]. Protective lncRNAs include DARS-AS1, MALAT1 (metastasis-associated lung adenocarcinoma transcript 1), and Miat (myocardial infarction associated transcript) [159,160,161]. In contrast, NEAT1 (nuclear paraspeckle assembly transcript 1), PRINS (psoriasis susceptibility-related RNA gene induced by stress), and LINC00520 are examples of pathogenic lncRNAs, which promote disease progression [148,162,163].

Details on the downstream mechanisms of each non-coding RNA are discussed elsewhere. Recent identification of numerous microRNAs suggests the feasibility of microRNA-based therapy using microRNA mimics or anti-microRNA oligonucleotides [51]. To date, no microRNAs have been tested in clinical trials for AKI-to-CKD transition; however, future studies may warrant therapeutic intervention with microRNAs.

### 3.6. Chromatin Conformational Changes

#### 3.6.1. High-Order Chromatin Architecture

To obtain an overall picture of the regulatory mechanism of gene expression, it is necessary to focus on the involvement of the high-order structure of chromatin. Within the nucleus, chromosomes occupy distinct territories and their radial locations are associated with gene content and activity [164]. Gene-poor, repressed regions are close to the nuclear periphery and are often in close contact with the nuclear lamina (NL) [165]. These transcriptionally silent, large genomic regions interact with the NL and are called lamina-associated domains (LADs) [166]. There are >1000 LADs distributed throughout the mammalian genome comprising about 35% of the genome [166,167]. Moreover, inactive chromatin compartments can aggregate near the nuclear chromocenter (pericentromere-associated domains; PADs) [168] and are localized to the nucleolar periphery (nucleolus associating domains; NADs) [164,169]. By contrast, gene-rich, activated regions are often localized centrally, and also at nuclear pore complexes [164].

In the lower scale, transcriptional regulation involves long-range interactions between regulatory elements, including enhancers and their target genes [164]. Enhancers are key regulatory DNA elements that control gene expression by engaging in physical contact with their target gene promoters, often over considerable genomic distances [170]. The recent advances in chromosome conformation capture (3C) and its derivatives, such as 4C, 5C, Hi-C (High-through chromosome conformation capture), and ChIA-PET (chromatin interaction analysis with paired-end tag sequencing), have enabled us to explore the detailed 3D architecture of the genome [171]. The 3D architecture may be organized into hierarchical layers, which are intended to represent structural and functional building units of genome organization, including topologically associating domains (TADs) and chromatin loops, often described as “insulated neighborhoods”, “loop domains”, and “CTCF contact domains” [170]. TADs are megabase-sized regions that confine the accessible space of regulatory elements, such as enhancers [164]. They are fundamental, structural, and functional units that are surrounded by boundary elements, including the insulator binding protein, CTCF (CCCTC-binding factor), across which the probability of chromatin interactions is reduced [164,172,173]. TADs are considered to function as microenvironments that promote intra-domain spatial interaction, thereby increasing the possibility of encounters among regulatory elements within the 3D nuclear space [170]. The latter, chromatin loops, are formed between two convergent CTCF regions that are coupled by cohesin and are believed to function as structural units of transcription [170]. They also raise the frequency of intra-domain interaction by confining enhancers to limited spatial domains, thereby activating gene expression [170]. These emerging local dynamics in chromatin conformation are important contributors to transcriptional regulation and may represent therapeutic targets.

#### 3.6.2. Chromatin Conformational Changes in AKI-to-CKD Transition

Brandt et al. revealed genome-wide alterations of the chromatin conformation in the TECs of fibrotic kidneys, focusing on CKD-associated susceptibility regions by conducting circular chromosome conformation capture (4C) analysis [174].

Wilflingseder et al. used ChIP-seq combined with RNA-seq of renal cortexes 2 days after IRI and identified several transcription factors that bound specifically to enhancer and super-enhancer sites [175]. They revealed enhancer dynamics and transcriptional changes during kidney repair. They also showed that the enhancer dynamics following AKI are dependent upon BRD4 in the kidney [175]. BRD4 is a member of the BET protein family and pharmacological inhibition results in therapeutic activity against various pathologies, such as cancer and inflammation [176]. BET protein family members interact with several key proteins involved in transcriptional regulation at enhancer sites [177]. They showed that BET inhibitors have different molecular effects in the kidney depending on the timing after injury [175], which suggests a therapeutic use for BET inhibitors with timely administration.

Crump et al. recently demonstrated that a decrease in BRD4 and mediator binding at the enhancers inhibited gene expression dramatically and rapidly; however, structures of enhancer–promoter looping remained stable, which suggests that the promotion of transcription and stabilization of enhancer–promoter interactions are separable events, and that the presence of an enhancer–promoter loop itself is not sufficient for maintaining transcription [178]. Further studies will elucidate the detailed mechanism of transcriptional regulation at this enhancer–promoter interaction level.

## 4. Future Perspectives

**Figure 6:**
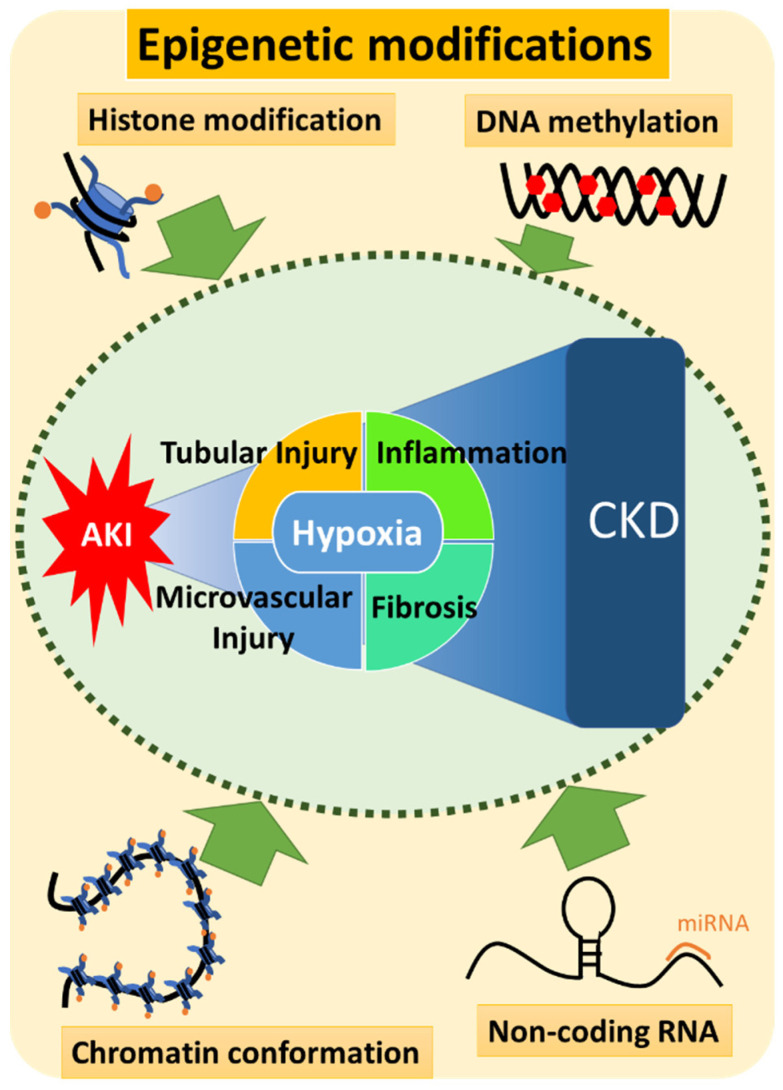
Epigenetic modifications contributing to the pathogenesis of AKI-to-CKD transition. Epigenetic modifications, including histone modification, DNA methylation, non-coding RNAs, and chromatin conformational changes, are considered to be largely involved in various pathologies contributing to AKI-to-CKD transition. Their expected contributions to the pathogenesis of AKI-to-CKD transition are supported by the existence of epigenetic memory; epigenetic alterations triggered by the initial injury can persist and predispose to the chronic fibrosis. Each epigenetic modification has a great potential as a novel therapeutic target for AKI-to-CKD transition.

In this review, we summarized the known pathophysiology of AKI-to-CKD transition and the current state of progress in the application of epigenetics to this process. Epigenetic alterations, including histone modification, DNA methylation, non-coding RNAs, and chromatin conformational changes, are largely involved in various pathologies contributing to the disease progression of AKI-to-CKD transition (Figure 6). Coupled with remarkable progress in various modern sequencing technologies, the field of epigenetics is making rapid progress. The continued production of an increasing body of evidence will clarify the detailed mechanisms of gene expression that are keys to elucidating the pathophysiology of various diseases. However, from the perspective of clinical applications, there are still many issues to be solved for epigenetic interventions including specificity. In particular, when treating non-cancer diseases, target specificity is one of the most important issues because of the issues of serious adverse effects and low efficacy of the intervention. To solve these problems, in addition to drug-side ingenuity, such as increasing target specificity and improving drug delivery, proper timing of the intervention is also an important factor to consider. As discussed above, AKI-to-CKD transition consists of some temporal phases and there are several temporal-specific gene expression patterns associated with the function of gene clusters, which enables us to improve target specificity by a timely specific approach to their epigenetic regulation mechanism. In addition, the time-course of AKI-to-CKD suggests the occurrence of epigenetic “memory” in the pathogenesis; thus, the first insult may have a long-term effect in the subsequent chronic phase, even after the recovery.

In the clinical setting, AKI is a very common community-acquired condition accompanied by various situations, such as volume depletion, sepsis, and chemotherapy with nephrotoxic agents, with a prevalence of up to 8 to 18% in hospitalized patients [81]. Currently, there are no effective therapies to intervene on the acquired predisposition to CKD progression for AKI survivors. Therapeutic agents that prevent the progression to CKD, which could be administered after suffering AKI or in the subsequent outpatient follow-up, would be very valuable and innovative.

AKI-to-CKD transition is considered to be a good target for epigenetic intervention. Further research will elucidate its detailed mechanism and lead to the development of novel therapeutic agents.

## Figures and Tables

**Table 1:** The effects of individual epigenetic agents on the pathophysiology of AKI-to-CKD transition. The targets of epigenetic drugs and their effects on kidney of animal models are listed.

Drug	Target	Model	Effects on Kidney	Ref.
Histone acetylation				
HDAC inhibitors				
TSA	Class Ι and ΙΙ HDAC inhibitor	IRI	↓ fibrosis	[123]
		UUO	↓ fibrosis	[124]
PTBA	Class Ι HDAC inhibitor	AA	↓ fibrosis, ↓ inflammation	[125]
PTBA analogs, UPHD 25	Class Ι HDAC inhibitor	IRI	↓ fibrosis, ↓ inflammation	[126]
FR276457	Class Ι and ΙΙ HDAC inhibitor	UUO	↓ fibrosis	[127]
Valproic acid	Class Ι HDAC inhibitor	IRI	↓ fibrosis, ↓ inflammation	[128]
MS-275	Class Ι HDAC inhibitor	LPS	↓ inflammation, ↑ renal function	[129]
		UUO	↓ fibrosis	[130]
		IRI	↓ fibrosis	[123]
		FA	↑ renal injury	[131]
TMP195	Class ΙΙ HDAC inhibitor	LPS	↓ inflammation, ↑ renal function	[132]
MC1568	Class ΙΙ HDAC inhibitor	UUO	↓ fibrosis, ↓ inflammation	[133]
RGFP966	Class Ι HDAC inhibitor	UUO/AA	↑ Klotho, ↓ fibrosis	[134]
ACY-1215	Class ΙΙ HDAC inhibitor	UUO	↓ fibrosis	[135]
Entinostat	Class Ι HDAC inhibitor	IRI	↑ renal function	[123,130]
		UUO	↓ fibrosis	
M4-PTB	Class Ι HDAC inhibitor	AA	↑ proliferation	[125,136]
		IRI	↓ fibrosis, ↓ inflammation	
HDAC (SIRT1) activators				
Resveratrol	SIRT1 activator	Cisplatin	↓ inflammation	[85]
		LPS	↓ inflammation	[86]
SRT-1720	SIRT1 activator	UUO	↓ fibrosis	[137]
		IRI	↑ proliferation, ↓ fibrosis	[87]
HAT inhibitors				
Curcumin	HAT p300/CBP inhibitor	Cisplatin	↓ inflammation	[138]
		LPS	↓ inflammation	[139]
		IRI	↓ fibrosis	[88]
Histone methylation				
HMT inhibitors				
DZNeP	HMT EZH2 inhibitor	UUO	↓ fibrosis	[140]
		IRI	↓ fibrosis	[100]
Sinefungin	HMT SET7/9 inhibitor	UUO	↓ fibrosis	[101]
BIX01294I	HMT G9a inhibitor	UUO	↓ fibrosis, ↑ Klotho	[102]
DNA methylation				
DNMT inhibitors				
5-azacytidine	DNMT inhibitor	UUO	↓ fibrosis	[116]
Decitabine	DNMT inhibitor	UUO	↓ fibrosis, ↑ Klotho	[141]
Hydralazine	induction of TET3	IRI	↓ fibrosis, ↑ renal function	[119]

TSA, trichostatin A; PTBA, phenylthiobutanoic acid; AA, aristolochic acid; LPS, lipopolysaccharide; FA, folic acid; downward arrows (↓), decreasing; upward arrows (↑), increasing.

## Data Availability

Date sharing is not applicable.

## References

[1] Romagnani P., Remuzzi G., Glassock R., Levin A., Jager K.J., Tonelli M., Massy Z., Wanner C., Anders H.J. (2017). Chronic kidney disease. Nat. Rev. Dis. Primers.

[2] Kuppe C., Ibrahim M.M., Kranz J., Zhang X., Ziegler S., Perales-Paton J., Jansen J., Reimer K.C., Smith J.R., Dobie R. (2021). Decoding myofibroblast origins in human kidney fibrosis. Nature.

[3] Nangaku M. (2004). Mechanisms of tubulointerstitial injury in the kidney: Final common pathways to end-stage renal failure. Intern. Med..

[4] Nangaku M. (2006). Chronic hypoxia and tubulointerstitial injury: A final common pathway to end-stage renal failure. J. Am. Soc. Nephrol..

[5] Jun J.I., Lau L.F. (2018). Resolution of organ fibrosis. J. Clin. Investig..

[6] Mimura I., Nangaku M. (2010). The suffocating kidney: Tubulointerstitial hypoxia in end-stage renal disease. Nat. Rev. Nephrol..

[7] Kellum J.A., Romagnani P., Ashuntantang G., Ronco C., Zarbock A., Anders H.J. (2021). Acute kidney injury. Nat. Rev. Dis. Primers.

[8] Mehta R.L., Burdmann E.A., Cerdá J., Feehally J., Finkelstein F., García-García G., Godin M., Jha V., Lameire N.H., Levin N.W. (2016). Recognition and management of acute kidney injury in the International Society of Nephrology 0by25 Global Snapshot: A multinational cross-sectional study. Lancet.

[9] Ullah M.M., Basile D.P. (2019). Role of Renal Hypoxia in the Progression from Acute Kidney Injury to Chronic Kidney Disease. Semin. Nephrol..

[10] Shu S., Wang Y., Zheng M., Liu Z., Cai J., Tang C., Dong Z. (2019). Hypoxia and Hypoxia-Inducible Factors in Kidney Injury and Repair. Cells.

[11] Loren P., Saavedra N., Saavedra K., Zambrano T., Moriel P., Salazar L.A. (2021). Epigenetic Mechanisms Involved in Cisplatin-Induced Nephrotoxicity: An Update. Pharmaceuticals.

[12] Nangaku M., Hirakawa Y., Mimura I., Inagi R., Tanaka T. (2017). Epigenetic Changes in the Acute Kidney Injury-to-Chronic Kidney Disease Transition. Nephron.

[13] Tanaka S., Tanaka T., Nangaku M. (2014). Hypoxia as a key player in the AKI-to-CKD transition. Am. J. Physiol. Ren. Physiol..

[14] Coca S.G., Singanamala S., Parikh C.R. (2012). Chronic kidney disease after acute kidney injury: A systematic review and meta-analysis. Kidney Int..

[15] See E.J., Jayasinghe K., Glassford N., Bailey M., Johnson D.W., Polkinghorne K.R., Toussaint N.D., Bellomo R. (2019). Long-term risk of adverse outcomes after acute kidney injury: A systematic review and meta-analysis of cohort studies using consensus definitions of exposure. Kidney Int..

[16] Basile D.P., Bonventre J.V., Mehta R., Nangaku M., Unwin R., Rosner M.H., Kellum J.A., Ronco C., Group A.X.W. (2016). Progression after AKI: Understanding Maladaptive Repair Processes to Predict and Identify Therapeutic Treatments. J. Am. Soc. Nephrol..

[17] Guzzi F., Cirillo L., Roperto R.M., Romagnani P., Lazzeri E. (2019). Molecular Mechanisms of the Acute Kidney Injury to Chronic Kidney Disease Transition: An Updated View. Int. J. Mol. Sci..

[18] Morgado-Pascual J.L., Marchant V., Rodrigues-Diez R., Dolade N., Suarez-Alvarez B., Kerr B., Valdivielso J.M., Ruiz-Ortega M., Rayego-Mateos S. (2018). Epigenetic Modification Mechanisms Involved in Inflammation and Fibrosis in Renal Pathology. Mediat. Inflamm..

[19] Chevalier R.L. (2016). The proximal tubule is the primary target of injury and progression of kidney disease: Role of the glomerulotubular junction. Am. J. Physiol. Ren. Physiol..

[20] Sato Y., Takahashi M., Yanagita M. (2020). Pathophysiology of AKI to CKD progression. Semin. Nephrol..

[21] Kumar S. (2018). Cellular and molecular pathways of renal repair after acute kidney injury. Kidney Int..

[22] Anders H.J. (2014). Immune system modulation of kidney regeneration—Mechanisms and implications. Nat. Rev. Nephrol..

[23] Henderson N.C., Rieder F., Wynn T.A. (2020). Fibrosis: From mechanisms to medicines. Nature.

[24] Kramann R., Wongboonsin J., Chang-Panesso M., Machado F.G., Humphreys B.D. (2017). Gli1^+^ Pericyte Loss Induces Capillary Rarefaction and Proximal Tubular Injury. J. Am. Soc. Nephrol..

[25] Sharfuddin A.A., Molitoris B.A. (2011). Pathophysiology of ischemic acute kidney injury. Nat. Rev. Nephrol..

[26] Linkermann A., Stockwell B.R., Krautwald S., Anders H.J. (2014). Regulated cell death and inflammation: An auto-amplification loop causes organ failure. Nat. Rev. Immunol..

[27] Mulay S.R., Linkermann A., Anders H.J. (2016). Necroinflammation in Kidney Disease. J. Am. Soc. Nephrol..

[28] Lazzeri E., Angelotti M.L., Conte C., Anders H.J., Romagnani P. (2019). Surviving Acute Organ Failure: Cell Polyploidization and Progenitor Proliferation. Trends Mol. Med..

[29] Lombardi D., Becherucci F., Romagnani P. (2016). How much can the tubule regenerate and who does it? An open question. Nephrol. Dial. Transplant..

[30] Kumar S., Liu J., Pang P., Krautzberger A.M., Reginensi A., Akiyama H., Schedl A., Humphreys B.D., McMahon A.P. (2015). Sox9 Activation Highlights a Cellular Pathway of Renal Repair in the Acutely Injured Mammalian Kidney. Cell Rep..

[31] Lazzeri E., Angelotti M.L., Peired A., Conte C., Marschner J.A., Maggi L., Mazzinghi B., Lombardi D., Melica M.E., Nardi S. (2018). Endocycle-related tubular cell hypertrophy and progenitor proliferation recover renal function after acute kidney injury. Nat. Commun..

[32] Shu Z., Row S., Deng W.M. (2018). Endoreplication: The Good, the Bad, and the Ugly. Trends Cell Biol..

[33] Liu B.C., Tang T.T., Lv L.L., Lan H.Y. (2018). Renal tubule injury: A driving force toward chronic kidney disease. Kidney Int..

[34] Canaud G., Bonventre J.V. (2015). Cell cycle arrest and the evolution of chronic kidney disease from acute kidney injury. Nephrol. Dial. Transplant..

[35] Liu J., Kumar S., Dolzhenko E., Alvarado G.F., Guo J., Lu C., Chen Y., Li M., Dessing M.C., Parvez R.K. (2017). Molecular characterization of the transition from acute to chronic kidney injury following ischemia/reperfusion. JCI Insight.

[36] Sharifian R., Okamura D.M., Denisenko O., Zager R.A., Johnson A., Gharib S.A., Bomsztyk K. (2018). Distinct patterns of transcriptional and epigenetic alterations characterize acute and chronic kidney injury. Sci. Rep..

[37] Cippa P.E., Sun B., Liu J., Chen L., Naesens M., McMahon A.P. (2018). Transcriptional trajectories of human kidney injury progression. JCI Insight.

[38] Kirita Y., Wu H., Uchimura K., Wilson P.C., Humphreys B.D. (2020). Cell profiling of mouse acute kidney injury reveals conserved cellular responses to injury. Proc. Natl. Acad. Sci. USA.

[39] Gerhardt L.M.S., Liu J., Koppitch K., Cippa P.E., McMahon A.P. (2021). Single-nuclear transcriptomics reveals diversity of proximal tubule cell states in a dynamic response to acute kidney injury. Proc. Natl. Acad. Sci. USA.

[40] Cao J., Cusanovich D.A., Ramani V., Aghamirzaie D., Pliner H.A., Hill A.J., Daza R.M., McFaline-Figueroa J.L., Packer J.S., Christiansen L. (2018). Joint profiling of chromatin accessibility and gene expression in thousands of single cells. Science.

[41] Muto Y., Wilson P.C., Ledru N., Wu H., Dimke H., Waikar S.S., Humphreys B.D. (2021). Single cell transcriptional and chromatin accessibility profiling redefine cellular heterogeneity in the adult human kidney. Nat. Commun..

[42] Govender M.A., Brandenburg J.T., Fabian J., Ramsay M. (2021). The Use of Omics for Diagnosing and Predicting Progression of Chronic Kidney Disease: A Scoping Review. Front. Genet..

[43] Wang H., Wang Y., Wang X., Huang H., Bao J., Zhong W., Li A. (2021). PTEN alleviates maladaptive repair of renal tubular epithelial cells by restoring CHMP2A-mediated phagosome closure. Cell Death Dis..

[44] Tran M.T., Zsengeller Z.K., Berg A.H., Khankin E.V., Bhasin M.K., Kim W., Clish C.B., Stillman I.E., Karumanchi S.A., Rhee E.P. (2016). PGC1α drives NAD biosynthesis linking oxidative metabolism to renal protection. Nature.

[45] Dubin R.F., Rhee E.P. (2020). Proteomics and Metabolomics in Kidney Disease, including Insights into Etiology, Treatment, and Prevention. Clin. J. Am. Soc. Nephrol..

[46] Li Z., Lu S., Li X. (2021). The role of metabolic reprogramming in tubular epithelial cells during the progression of acute kidney injury. Cell. Mol. Life Sci..

[47] Morigi M., Perico L., Benigni A. (2018). Sirtuins in Renal Health and Disease. J. Am. Soc. Nephrol..

[48] Wang Q., Xu J., Li X., Liu Z., Han Y., Xu X., Li X., Tang Y., Liu Y., Yu T. (2019). Sirt3 modulate renal ischemia-reperfusion injury through enhancing mitochondrial fusion and activating the ERK-OPA1 signaling pathway. J. Cell. Physiol..

[49] Li W., Sun Z. (2019). Mechanism of Action for HDAC Inhibitors-Insights from Omics Approaches. Int. J. Mol. Sci..

[50] Egger G., Liang G., Aparicio A., Jones P.A. (2004). Epigenetics in human disease and prospects for epigenetic therapy. Nature.

[51] Guo C., Dong G., Liang X., Dong Z. (2019). Epigenetic regulation in AKI and kidney repair: Mechanisms and therapeutic implications. Nat. Rev. Nephrol..

[52] Ding H., Zhang L., Yang Q., Zhang X., Li X. (2021). Epigenetics in kidney diseases. Adv. Clin. Chem..

[53] Mimura I., Nangaku M., Kanki Y., Tsutsumi S., Inoue T., Kohro T., Yamamoto S., Fujita T., Shimamura T., Suehiro J. (2012). Dynamic change of chromatin conformation in response to hypoxia enhances the expression of GLUT3 (SLC2A3) by cooperative interaction of hypoxia-inducible factor 1 and KDM3A. Mol. Cell. Biol..

[54] Wanner N., Bechtel-Walz W. (2017). Epigenetics of kidney disease. Cell Tissue Res..

[55] Prachayasittikul V., Prathipati P., Pratiwi R., Phanus-Umporn C., Malik A.A., Schaduangrat N., Seenprachawong K., Wongchitrat P., Supokawej A., Prachayasittikul V. (2017). Exploring the epigenetic drug discovery landscape. Expert Opin. Drug Discov..

[56] Wilson P.C., Ledru N., Humphreys B.D. (2020). Epigenomics and the kidney. Curr. Opin. Nephrol. Hypertens..

[57] Tampe B., Zeisberg M. (2018). Chromatin dynamics at the core of kidney fibrosis. Matrix Biol..

[58] Yamanaka S., Nishihara H., Toh H., Eijy Nagai L.A., Hashimoto K., Park S.J., Shibuya A., Suzuki A.M., Tanaka Y., Nakai K. (2019). Broad Heterochromatic Domains Open in Gonocyte Development Prior to De Novo DNA Methylation. Dev. Cell.

[59] Stricker S.H., Koferle A., Beck S. (2017). From profiles to function in epigenomics. Nat. Rev. Genet..

[60] Tanaka T. (2018). Epigenetic changes mediating transition to chronic kidney disease: Hypoxic memory. Acta Physiol..

[61] Mimura I., Tanaka T., Nangaku M. (2013). Novel therapeutic strategy with hypoxia-inducible factors via reversible epigenetic regulation mechanisms in progressive tubulointerstitial fibrosis. Semin. Nephrol..

[62] Mimura I., Tanaka T., Wada Y., Kodama T., Nangaku M. (2011). Pathophysiological response to hypoxia—From the molecular mechanisms of malady to drug discovery: Epigenetic regulation of the hypoxic response via hypoxia-inducible factor and histone modifying enzymes. J. Pharmacol. Sci..

[63] Mimura I., Tanaka T., Nangaku M. (2016). New insights into molecular mechanisms of epigenetic regulation in kidney disease. Clin. Exp. Pharmacol. Physiol..

[64] Mimura I., Hirakawa Y., Kanki Y., Kushida N., Nakaki R., Suzuki Y., Tanaka T., Aburatani H., Nangaku M. (2017). Novel lnc RNA regulated by HIF-1 inhibits apoptotic cell death in the renal tubular epithelial cells under hypoxia. Physiol. Rep..

[65] Mimura I., Kanki Y., Kodama T., Nangaku M. (2014). Revolution of nephrology research by deep sequencing: ChIP-seq and RNA-seq. Kidney Int..

[66] Shahbazian M.D., Grunstein M. (2007). Functions of site-specific histone acetylation and deacetylation. Annu. Rev. Biochem..

[67] Haberland M., Montgomery R.L., Olson E.N. (2009). The many roles of histone deacetylases in development and physiology: Implications for disease and therapy. Nat. Rev. Genet..

[68] Hodawadekar S.C., Marmorstein R. (2007). Chemistry of acetyl transfer by histone modifying enzymes: Structure, mechanism and implications for effector design. Oncogene.

[69] Filippakopoulos P., Knapp S. (2014). Targeting bromodomains: Epigenetic readers of lysine acetylation. Nat. Rev. Drug Discov..

[70] Belkina A.C., Denis G.V. (2012). BET domain co-regulators in obesity, inflammation and cancer. Nat. Rev. Cancer.

[71] Fontecha-Barriuso M., Martin-Sanchez D., Ruiz-Andres O., Poveda J., Sanchez-Nino M.D., Valino-Rivas L., Ruiz-Ortega M., Ortiz A., Sanz A.B. (2018). Targeting epigenetic DNA and histone modifications to treat kidney disease. Nephrol. Dial. Transplant..

[72] Fu L.L., Tian M., Li X., Li J.J., Huang J., Ouyang L., Zhang Y., Liu B. (2015). Inhibition of BET bromodomains as a therapeutic strategy for cancer drug discovery. Oncotarget.

[73] Jenuwein T., Allis C.D. (2001). Translating the histone code. Science.

[74] Marumo T., Hishikawa K., Yoshikawa M., Fujita T. (2008). Epigenetic regulation of BMP7 in the regenerative response to ischemia. J. Am. Soc. Nephrol..

[75] Ruiz-Andres O., Suarez-Alvarez B., Sánchez-Ramos C., Monsalve M., Sanchez-Niño M.D., Ruiz-Ortega M., Egido J., Ortiz A., Sanz A.B. (2016). The inflammatory cytokine TWEAK decreases PGC-1α expression and mitochondrial function in acute kidney injury. Kidney Int..

[76] Moreno J.A., Izquierdo M.C., Sanchez-Niño M.D., Suárez-Alvarez B., Lopez-Larrea C., Jakubowski A., Blanco J., Ramirez R., Selgas R., Ruiz-Ortega M. (2011). The inflammatory cytokines TWEAK and TNFα reduce renal klotho expression through NFκB. J. Am. Soc. Nephrol..

[77] Zager R.A., Johnson A.C., Becker K. (2011). Acute unilateral ischemic renal injury induces progressive renal inflammation, lipid accumulation, histone modification, and “end-stage” kidney disease. Am. J. Physiol. Ren. Physiol..

[78] Hewitson T.D., Holt S.G., Tan S.J., Wigg B., Samuel C.S., Smith E.R. (2017). Epigenetic Modifications to H3K9 in Renal Tubulointerstitial Cells after Unilateral Ureteric Obstruction and TGF-β1 Stimulation. Front. Pharmacol..

[79] Huang J., Wan D., Li J., Chen H., Huang K., Zheng L. (2015). Histone acetyltransferase PCAF regulates inflammatory molecules in the development of renal injury. Epigenetics.

[80] Mar D., Gharib S.A., Zager R.A., Johnson A., Denisenko O., Bomsztyk K. (2015). Heterogeneity of epigenetic changes at ischemia/reperfusion- and endotoxin-induced acute kidney injury genes. Kidney Int..

[81] Zhou X., Chen H., Shi Y., Ma X., Zhuang S., Liu N. (2021). The Role and Mechanism of Histone Deacetylases in Acute Kidney Injury. Front. Pharmacol..

[82] Kim J.I., Jung K.J., Jang H.S., Park K.M. (2013). Gender-specific role of HDAC11 in kidney ischemia- and reperfusion-induced PAI-1 expression and injury. Am. J. Physiol. Ren. Physiol..

[83] Liu N., Zhuang S. (2015). Treatment of chronic kidney diseases with histone deacetylase inhibitors. Front. Physiol..

[84] Hyndman K.A., Speed J.S., Mendoza L.D., Allan J.M., Colson J., Sedaka R., Jin C., Jung H.J., El-Dahr S., Pollock D.M. (2020). Fluid-electrolyte homeostasis requires histone deacetylase function. JCI Insight.

[85] Do Amaral C.L., Francescato H.D., Coimbra T.M., Costa R.S., Darin J.D., Antunes L.M., Bianchi Mde L. (2008). Resveratrol attenuates cisplatin-induced nephrotoxicity in rats. Arch. Toxicol..

[86] Xu S., Gao Y., Zhang Q., Wei S., Chen Z., Dai X., Zeng Z., Zhao K.S. (2016). SIRT1/3 Activation by Resveratrol Attenuates Acute Kidney Injury in a Septic Rat Model. Oxid. Med. Cell. Longev..

[87] Fan H., Yang H.C., You L., Wang Y.Y., He W.J., Hao C.M. (2013). The histone deacetylase, SIRT1, contributes to the resistance of young mice to ischemia/reperfusion-induced acute kidney injury. Kidney Int..

[88] Bayrak O., Uz E., Bayrak R., Turgut F., Atmaca A.F., Sahin S., Yildirim M.E., Kaya A., Cimentepe E., Akcay A. (2008). Curcumin protects against ischemia/reperfusion injury in rat kidneys. World J. Urol..

[89] Kasi P.D., Tamilselvam R., Skalicka-Woźniak K., Nabavi S.F., Daglia M., Bishayee A., Pazoki-Toroudi H., Nabavi S.M. (2016). Molecular targets of curcumin for cancer therapy: An updated review. Tumour Biol..

[90] Black J.C., Van Rechem C., Whetstine J.R. (2012). Histone lysine methylation dynamics: Establishment, regulation, and biological impact. Mol. Cell.

[91] Hyun K., Jeon J., Park K., Kim J. (2017). Writing, erasing and reading histone lysine methylations. Exp. Mol. Med..

[92] Rea S., Eisenhaber F., O’Carroll D., Strahl B.D., Sun Z.W., Schmid M., Opravil S., Mechtler K., Ponting C.P., Allis C.D. (2000). Regulation of chromatin structure by site-specific histone H3 methyltransferases. Nature.

[93] Dambacher S., Hahn M., Schotta G. (2010). Epigenetic regulation of development by histone lysine methylation. Heredity.

[94] Zhou X., Zang X., Ponnusamy M., Masucci M.V., Tolbert E., Gong R., Zhao T.C., Liu N., Bayliss G., Dworkin L.D. (2016). Enhancer of Zeste Homolog 2 Inhibition Attenuates Renal Fibrosis by Maintaining Smad7 and Phosphatase and Tensin Homolog Expression. J. Am. Soc. Nephrol..

[95] Li Z., Li N. (2021). Epigenetic Modification Drives Acute Kidney Injury-to-Chronic Kidney Disease Progression. Nephron.

[96] Naito M., Bomsztyk K., Zager R.A. (2008). Endotoxin mediates recruitment of RNA polymerase II to target genes in acute renal failure. J. Am. Soc. Nephrol..

[97] Zager R.A., Johnson A.C. (2009). Renal ischemia-reperfusion injury upregulates histone-modifying enzyme systems and alters histone expression at proinflammatory/profibrotic genes. Am. J. Physiol. Ren. Physiol..

[98] Naito M., Bomsztyk K., Zager R.A. (2009). Renal ischemia-induced cholesterol loading: Transcription factor recruitment and chromatin remodeling along the HMG CoA reductase gene. Am. J. Pathol..

[99] Johnson A.C., Ware L.B., Himmelfarb J., Zager R.A. (2011). HMG-CoA reductase activation and urinary pellet cholesterol elevations in acute kidney injury. Clin. J. Am. Soc. Nephrol..

[100] Mimura I., Hirakawa Y., Kanki Y., Nakaki R., Suzuki Y., Tanaka T., Aburatani H., Nangaku M. (2018). Genome-wide analysis revealed that DZNep reduces tubulointerstitial fibrosis via down-regulation of pro-fibrotic genes. Sci. Rep..

[101] Sasaki K., Doi S., Nakashima A., Irifuku T., Yamada K., Kokoroishi K., Ueno T., Doi T., Hida E., Arihiro K. (2016). Inhibition of SET Domain-Containing Lysine Methyltransferase 7/9 Ameliorates Renal Fibrosis. J. Am. Soc. Nephrol..

[102] Irifuku T., Doi S., Sasaki K., Doi T., Nakashima A., Ueno T., Yamada K., Arihiro K., Kohno N., Masaki T. (2016). Inhibition of H3K9 histone methyltransferase G9a attenuates renal fibrosis and retains klotho expression. Kidney Int..

[103] Xu H., Wu M., Ma X., Huang W., Xu Y. (2021). Function and Mechanism of Novel Histone Posttranslational Modifications in Health and Disease. Biomed. Res. Int..

[104] Tan M., Luo H., Lee S., Jin F., Yang J.S., Montellier E., Buchou T., Cheng Z., Rousseaux S., Rajagopal N. (2011). Identification of 67 histone marks and histone lysine crotonylation as a new type of histone modification. Cell.

[105] Ruiz-Andres O., Sanchez-Niño M.D., Cannata-Ortiz P., Ruiz-Ortega M., Egido J., Ortiz A., Sanz A.B. (2016). Histone lysine crotonylation during acute kidney injury in mice. Dis. Model. Mech..

[106] Wan J., Liu H., Chu J., Zhang H. (2019). Functions and mechanisms of lysine crotonylation. J. Cell. Mol. Med..

[107] Hellman A., Chess A. (2007). Gene body-specific methylation on the active X chromosome. Science.

[108] Greenberg M.V.C., Bourc’his D. (2019). The diverse roles of DNA methylation in mammalian development and disease. Nat. Rev. Mol. Cell Biol..

[109] Robertson K.D. (2001). DNA methylation, methyltransferases, and cancer. Oncogene.

[110] Guo X., Wang L., Li J., Ding Z., Xiao J., Yin X., He S., Shi P., Dong L., Li G. (2015). Structural insight into autoinhibition and histone H3-induced activation of DNMT3A. Nature.

[111] Piunti A., Shilatifard A. (2016). Epigenetic balance of gene expression by Polycomb and COMPASS families. Science.

[112] Ishiyama S., Nishiyama A., Saeki Y., Moritsugu K., Morimoto D., Yamaguchi L., Arai N., Matsumura R., Kawakami T., Mishima Y. (2017). Structure of the Dnmt1 Reader Module Complexed with a Unique Two-Mono-Ubiquitin Mark on Histone H3 Reveals the Basis for DNA Methylation Maintenance. Mol. Cell.

[113] Wu X., Zhang Y. (2017). TET-mediated active DNA demethylation: Mechanism, function and beyond. Nat. Rev. Genet..

[114] Huang N., Tan L., Xue Z., Cang J., Wang H. (2012). Reduction of DNA hydroxymethylation in the mouse kidney insulted by ischemia reperfusion. Biochem. Biophys. Res. Commun..

[115] Zhao Y., Ding C., Xue W., Ding X., Zheng J., Gao Y., Xia X., Li S., Liu J., Han F. (2017). Genome-wide DNA methylation analysis in renal ischemia reperfusion injury. Gene.

[116] Chang Y.T., Yang C.C., Pan S.Y., Chou Y.H., Chang F.C., Lai C.F., Tsai M.H., Hsu H.L., Lin C.H., Chiang W.C. (2016). DNA methyltransferase inhibition restores erythropoietin production in fibrotic murine kidneys. J. Clin. Investig..

[117] Zhang C., Liang Y., Lei L., Zhu G., Chen X., Jin T., Wu Q. (2013). Hypermethylations of RASAL1 and KLOTHO is associated with renal dysfunction in a Chinese population environmentally exposed to cadmium. Toxicol. Appl. Pharmacol..

[118] Xu X., Tan X., Tampe B., Wilhelmi T., Hulshoff M.S., Saito S., Moser T., Kalluri R., Hasenfuss G., Zeisberg E.M. (2018). High-fidelity CRISPR/Cas9- based gene-specific hydroxymethylation rescues gene expression and attenuates renal fibrosis. Nat. Commun..

[119] Tampe B., Steinle U., Tampe D., Carstens J.L., Korsten P., Zeisberg E.M., Muller G.A., Kalluri R., Zeisberg M. (2017). Low-dose hydralazine prevents fibrosis in a murine model of acute kidney injury-to-chronic kidney disease progression. Kidney Int..

[120] Bechtel W., McGoohan S., Zeisberg E.M., Müller G.A., Kalbacher H., Salant D.J., Müller C.A., Kalluri R., Zeisberg M. (2010). Methylation determines fibroblast activation and fibrogenesis in the kidney. Nat. Med..

[121] Kandler M.R., Mah G.T., Tejani A.M., Stabler S.N., Salzwedel D.M. (2011). Hydralazine for essential hypertension. Cochrane Database Syst. Rev..

[122] Larkin B.P., Glastras S.J., Chen H., Pollock C.A., Saad S. (2018). DNA methylation and the potential role of demethylating agents in prevention of progressive chronic kidney disease. FASEB J..

[123] Levine M.H., Wang Z., Bhatti T.R., Wang Y., Aufhauser D.D., McNeal S., Liu Y., Cheraghlou S., Han R., Wang L. (2015). Class-specific histone/protein deacetylase inhibition protects against renal ischemia reperfusion injury and fibrosis formation. Am. J. Transplant..

[124] Manson S.R., Song J.B., Hruska K.A., Austin P.F. (2014). HDAC dependent transcriptional repression of Bmp-7 potentiates TGF-β mediated renal fibrosis in obstructive uropathy. J. Urol..

[125] Novitskaya T., McDermott L., Zhang K.X., Chiba T., Paueksakon P., Hukriede N.A., de Caestecker M.P. (2014). A PTBA small molecule enhances recovery and reduces postinjury fibrosis after aristolochic acid-induced kidney injury. Am. J. Physiol. Ren. Physiol..

[126] Skrypnyk N.I., Sanker S., Skvarca L.B., Novitskaya T., Woods C., Chiba T., Patel K., Goldberg N.D., McDermott L., Vinson P.N. (2016). Delayed treatment with PTBA analogs reduces postinjury renal fibrosis after kidney injury. Am. J. Physiol. Ren. Physiol..

[127] Kinugasa F., Noto T., Matsuoka H., Urano Y., Sudo Y., Takakura S., Mutoh S. (2010). Prevention of renal interstitial fibrosis via histone deacetylase inhibition in rats with unilateral ureteral obstruction. Transpl. Immunol..

[128] Costalonga E.C., Silva F.M., Noronha I.L. (2016). Valproic Acid Prevents Renal Dysfunction and Inflammation in the Ischemia-Reperfusion Injury Model. Biomed. Res. Int..

[129] Zhang H., Zhang W., Jiao F., Li X., Zhang H., Wang L., Gong Z. (2018). The Nephroprotective Effect of MS-275 on Lipopolysaccharide (LPS)-Induced Acute Kidney Injury by Inhibiting Reactive Oxygen Species (ROS)-Oxidative Stress and Endoplasmic Reticulum Stress. Med. Sci. Monit..

[130] Liu N., He S., Ma L., Ponnusamy M., Tang J., Tolbert E., Bayliss G., Zhao T.C., Yan H., Zhuang S. (2013). Blocking the class I histone deacetylase ameliorates renal fibrosis and inhibits renal fibroblast activation via modulating TGF-beta and EGFR signaling. PLoS ONE.

[131] Tang J., Yan Y., Zhao T.C., Gong R., Bayliss G., Yan H., Zhuang S. (2014). Class I HDAC activity is required for renal protection and regeneration after acute kidney injury. Am. J. Physiol. Ren. Physiol..

[132] Xiong C., Guan Y., Zhou X., Liu L., Zhuang M.A., Zhang W., Zhang Y., Masucci M.V., Bayliss G., Zhao T.C. (2019). Selective inhibition of class IIa histone deacetylases alleviates renal fibrosis. FASEB J..

[133] Zhang W., Guan Y., Bayliss G., Zhuang S. (2020). Class IIa HDAC inhibitor TMP195 alleviates lipopolysaccharide-induced acute kidney injury. Am. J. Physiol. Ren. Physiol..

[134] Chen F., Gao Q., Wei A., Chen X., Shi Y., Wang H., Cao W. (2021). Histone deacetylase 3 aberration inhibits Klotho transcription and promotes renal fibrosis. Cell Death Differ..

[135] Chen X., Yu C., Hou X., Li J., Li T., Qiu A., Liu N., Zhuang S. (2020). Histone deacetylase 6 inhibition mitigates renal fibrosis by suppressing TGF-β and EGFR signaling pathways in obstructive nephropathy. Am. J. Physiol. Ren. Physiol..

[136] Cianciolo Cosentino C., Skrypnyk N.I., Brilli L.L., Chiba T., Novitskaya T., Woods C., West J., Korotchenko V.N., McDermott L., Day B.W. (2013). Histone deacetylase inhibitor enhances recovery after AKI. J. Am. Soc. Nephrol..

[137] He W., Wang Y., Zhang M.Z., You L., Davis L.S., Fan H., Yang H.C., Fogo A.B., Zent R., Harris R.C. (2010). Sirt1 activation protects the mouse renal medulla from oxidative injury. J. Clin. Investig..

[138] Kuhad A., Pilkhwal S., Sharma S., Tirkey N., Chopra K. (2007). Effect of curcumin on inflammation and oxidative stress in cisplatin-induced experimental nephrotoxicity. J. Agric. Food Chem..

[139] Memis D., Hekimoglu S., Sezer A., Altaner S., Sut N., Usta U. (2008). Curcumin attenuates the organ dysfunction caused by endotoxemia in the rat. Nutrition.

[140] Liang H., Huang Q., Liao M.J., Xu F., Zhang T., He J., Zhang L., Liu H.Z. (2019). EZH2 plays a crucial role in ischemia/reperfusion-induced acute kidney injury by regulating p38 signaling. Inflamm. Res..

[141] Yin S., Zhang Q., Yang J., Lin W., Li Y., Chen F., Cao W. (2017). TGFβ-incurred epigenetic aberrations of miRNA and DNA methyltransferase suppress Klotho and potentiate renal fibrosis. Biochim. Biophys. Acta Mol. Cell Res..

[142] Kota S.K., Kota S.B. (2017). Noncoding RNA and epigenetic gene regulation in renal diseases. Drug Discov. Today.

[143] Fan Y., Chen H., Huang Z., Zheng H., Zhou J. (2020). Emerging role of miRNAs in renal fibrosis. RNA Biol..

[144] Carthew R.W., Sontheimer E.J. (2009). Origins and Mechanisms of miRNAs and siRNAs. Cell.

[145] Brennecke J., Malone C.D., Aravin A.A., Sachidanandam R., Stark A., Hannon G.J. (2008). An epigenetic role for maternally inherited piRNAs in transposon silencing. Science.

[146] Mercer T.R., Mattick J.S. (2013). Structure and function of long noncoding RNAs in epigenetic regulation. Nat. Struct. Mol. Biol..

[147] Wang K.C., Chang H.Y. (2011). Molecular mechanisms of long noncoding RNAs. Mol. Cell.

[148] Jiang X., Li D., Shen W., Shen X., Liu Y. (2019). LncRNA NEAT1 promotes hypoxia-induced renal tubular epithelial apoptosis through downregulating miR-27a-3p. J. Cell. Biochem..

[149] Bijkerk R., van Solingen C., de Boer H.C., van der Pol P., Khairoun M., de Bruin R.G., van Oeveren-Rietdijk A.M., Lievers E., Schlagwein N., van Gijlswijk D.J. (2014). Hematopoietic microRNA-126 protects against renal ischemia/reperfusion injury by promoting vascular integrity. J. Am. Soc. Nephrol..

[150] Hao J., Wei Q., Mei S., Li L., Su Y., Mei C., Dong Z. (2017). Induction of microRNA-17-5p by p53 protects against renal ischemia-reperfusion injury by targeting death receptor 6. Kidney Int..

[151] Wei Q., Sun H., Song S., Liu Y., Liu P., Livingston M.J., Wang J., Liang M., Mi Q.S., Huo Y. (2018). MicroRNA-668 represses MTP18 to preserve mitochondrial dynamics in ischemic acute kidney injury. J. Clin. Investig..

[152] Chen W., Ruan Y., Zhao S., Ning J., Rao T., Yu W., Zhou X., Liu C., Qi Y., Cheng F. (2019). MicroRNA-205 inhibits the apoptosis of renal tubular epithelial cells via the PTEN/Akt pathway in renal ischemia-reperfusion injury. Am. J. Transl. Res..

[153] Lorenzen J.M., Kaucsar T., Schauerte C., Schmitt R., Rong S., Hübner A., Scherf K., Fiedler J., Martino F., Kumarswamy R. (2014). MicroRNA-24 antagonism prevents renal ischemia reperfusion injury. J. Am. Soc. Nephrol..

[154] Bhatt K., Wei Q., Pabla N., Dong G., Mi Q.S., Liang M., Mei C., Dong Z. (2015). MicroRNA-687 Induced by Hypoxia-Inducible Factor-1 Targets Phosphatase and Tensin Homolog in Renal Ischemia-Reperfusion Injury. J. Am. Soc. Nephrol..

[155] Yuan J., Benway C.J., Bagley J., Iacomini J. (2015). MicroRNA-494 promotes cyclosporine-induced nephrotoxicity and epithelial to mesenchymal transition by inhibiting PTEN. Am. J. Transplant..

[156] Guan H., Peng R., Mao L., Fang F., Xu B., Chen M. (2020). Injured tubular epithelial cells activate fibroblasts to promote kidney fibrosis through miR-150-containing exosomes. Exp. Cell Res..

[157] Huang S.J., Huang J., Yan Y.B., Qiu J., Tan R.Q., Liu Y., Tian Q., Guan L., Niu S.S., Zhang Y. (2020). The renoprotective effect of curcumin against cisplatin-induced acute kidney injury in mice: Involvement of miR-181a/PTEN axis. Ren. Fail..

[158] Lv W., Fan F., Wang Y., Gonzalez-Fernandez E., Wang C., Yang L., Booz G.W., Roman R.J. (2018). Therapeutic potential of microRNAs for the treatment of renal fibrosis and CKD. Physiol. Genom..

[159] Chen H., Fan Y., Jing H., Tang S., Zhou J. (2020). Emerging role of lncRNAs in renal fibrosis. Arch. Biochem. Biophys..

[160] Tian H., Wu M., Zhou P., Huang C., Ye C., Wang L. (2018). The long non-coding RNA MALAT1 is increased in renal ischemia-reperfusion injury and inhibits hypoxia-induced inflammation. Ren. Fail..

[161] Bijkerk R., Au Y.W., Stam W., Duijs J., Koudijs A., Lievers E., Rabelink T.J., van Zonneveld A.J. (2019). Long Non-coding RNAs Rian and Miat Mediate Myofibroblast Formation in Kidney Fibrosis. Front. Pharmacol..

[162] Yu T.M., Palanisamy K., Sun K.T., Day Y.J., Shu K.H., Wang I.K., Shyu W.C., Chen P., Chen Y.L., Li C.Y. (2016). RANTES mediates kidney ischemia reperfusion injury through a possible role of HIF-1α and LncRNA PRINS. Sci. Rep..

[163] Tian X., Ji Y., Liang Y., Zhang J., Guan L., Wang C. (2019). LINC00520 targeting miR-27b-3p regulates OSMR expression level to promote acute kidney injury development through the PI3K/AKT signaling pathway. J. Cell. Physiol..

[164] Vermunt M.W., Zhang D., Blobel G.A. (2019). The interdependence of gene-regulatory elements and the 3D genome. J. Cell Biol..

[165] van Steensel B., Belmont A.S. (2017). Lamina-Associated Domains: Links with Chromosome Architecture, Heterochromatin, and Gene Repression. Cell.

[166] Yanez-Cuna J.O., van Steensel B. (2017). Genome-nuclear lamina interactions: From cell populations to single cells. Curr. Opin. Genet. Dev..

[167] Guelen L., Pagie L., Brasset E., Meuleman W., Faza M.B., Talhout W., Eussen B.H., de Klein A., Wessels L., de Laat W. (2008). Domain organization of human chromosomes revealed by mapping of nuclear lamina interactions. Nature.

[168] Wijchers P.J., Geeven G., Eyres M., Bergsma A.J., Janssen M., Verstegen M., Zhu Y., Schell Y., Vermeulen C., de Wit E. (2015). Characterization and dynamics of pericentromere-associated domains in mice. Genome Res..

[169] van Koningsbruggen S., Gierlinski M., Schofield P., Martin D., Barton G.J., Ariyurek Y., den Dunnen J.T., Lamond A.I. (2010). High-resolution whole-genome sequencing reveals that specific chromatin domains from most human chromosomes associate with nucleoli. Mol. Biol. Cell.

[170] Schoenfelder S., Fraser P. (2019). Long-range enhancer-promoter contacts in gene expression control. Nat. Rev. Genet..

[171] Lupianez D.G., Spielmann M., Mundlos S. (2016). Breaking TADs: How Alterations of Chromatin Domains Result in Disease. Trends Genet..

[172] Fudenberg G., Imakaev M., Lu C., Goloborodko A., Abdennur N., Mirny L.A. (2016). Formation of Chromosomal Domains by Loop Extrusion. Cell Rep..

[173] Dixon J.R., Selvaraj S., Yue F., Kim A., Li Y., Shen Y., Hu M., Liu J.S., Ren B. (2012). Topological domains in mammalian genomes identified by analysis of chromatin interactions. Nature.

[174] Brandt M.M., Meddens C.A., Louzao-Martinez L., van den Dungen N.A.M., Lansu N.R., Nieuwenhuis E.E.S., Duncker D.J., Verhaar M.C., Joles J.A., Mokry M. (2018). Chromatin Conformation Links Distal Target Genes to CKD Loci. J. Am. Soc. Nephrol..

[175] Wilflingseder J., Willi M., Lee H.K., Olauson H., Jankowski J., Ichimura T., Erben R., Valerius M.T., Hennighausen L., Bonventre J.V. (2020). Enhancer and super-enhancer dynamics in repair after ischemic acute kidney injury. Nat. Commun..

[176] De Raedt T., Beert E., Pasmant E., Luscan A., Brems H., Ortonne N., Helin K., Hornick J.L., Mautner V., Kehrer-Sawatzki H. (2014). PRC2 loss amplifies Ras-driven transcription and confers sensitivity to BRD4-based therapies. Nature.

[177] Jung M., Philpott M., Muller S., Schulze J., Badock V., Eberspacher U., Moosmayer D., Bader B., Schmees N., Fernandez-Montalvan A. (2014). Affinity map of bromodomain protein 4 (BRD4) interactions with the histone H4 tail and the small molecule inhibitor JQ1. J. Biol. Chem..

[178] Crump N.T., Ballabio E., Godfrey L., Thorne R., Repapi E., Kerry J., Tapia M., Hua P., Lagerholm C., Filippakopoulos P. (2021). BET inhibition disrupts transcription but retains enhancer-promoter contact. Nat. Commun..

